# Benefit and Adherence of the Disease Management Program “Diabetes 2”: A Comparison of Turkish Immigrants and German Natives with Diabetes

**DOI:** 10.3390/ijerph110909723

**Published:** 2014-09-17

**Authors:** Anna Christin Makowski, Christopher Kofahl

**Affiliations:** Department of Medical Sociology, University Medical Center, Hamburg-Eppendorf, Martinistraße 52, 20246 Hamburg, Germany; E-Mail: kofahl@uke.de

**Keywords:** diabetes type 2, disease management program, equity, quality of care, migrants, Turkey, Germany

## Abstract

There is an ongoing debate about equity and equality in health care, and whether immigrants benefit equally from services as the non-immigrant population. The study focuses on benefits from and adherence to the diabetes mellitus type 2 (DM 2) disease management program (DMP) among Turkish immigrants in Germany. So far, it has not been researched whether this group benefits from enrollment in the DMP as well as diabetics from the non-immigrant population. Data on the non-immigrant sample (N = 702) stem from a survey among members of a German health insurance, the Turkish immigrant sample (N = 102) was recruited in the area of Hamburg. Identical questions in both surveys enable comparing major components. Regarding process quality, Turkish diabetics do not differ from the non-immigrant sample; moreover, they have significantly more often received documentation and diabetes training. In terms of outcome quality however, results display a greater benefit on behalf of the non-immigrant sample (e.g., blood parameters and body mass index), and they also met more of the DMP criteria. This underlines the need of diabetics with Turkish background for further education and information in order to become the empowered patient as is intended by the DMP as well as to prevent comorbidities.

## 1. Introduction

Over the last years, ethnical and cultural diversity has become an important issue in public health and health care research [1]. Accrediting that health systems need to take measures to adapt to migrants’ specific needs led to growing international attention to migrant health policy [2,3]. The health of migrants can be determined by several factors related to the migration process itself. However, migrants usually suffer from health problems similar to those of the population in the host country, and the discussion on migrant health is focusing more and more on issues of equity and equality [4]. There is an ongoing debate whether immigrants access health care services appropriately, and whether they benefit from services in the same way as the non-immigrant population [5,6].

The present study addresses the latter aspect, focusing on one of the biggest immigrant populations in Germany. In the framework of the project “Health Literacy of Turkish Diabetics” [7,8], 294 Turkish immigrants with diabetes mellitus type 2 (DM 2) were interviewed about their well-being and health, their health literacy, beliefs and attitudes, their socio-economical background and social networks, but also about their individual health care, specifically with respect for the “Diabetes 2” disease management program (DMP). In particular, we wanted to know to what extent Turkish diabetics benefit from enrollment in the DMP compared to a sample of non-immigrant diabetics who were enrolled in the Diabetes-2-DMP as well. For this comparison we could refer to a doctoral thesis [9] by Barbara Ruß-Thiel, who addressed the topic of diabetes care and DMP among the non-immigrant population. However, due to data protection and rights restrictions, we were not allowed to access the raw data of the study on German diabetics. For the sample of Turkish migrants we could use all data available. Despite this limitation, the use of identical questions in both studies allows comparisons up to a certain degree.

In 2002, the German Health Care Reform Act defined a process for introducing DMPs for chronic conditions such as diabetes, asthma or breast cancer. The quality of care was expected to improve within the DMPs by means of guidelines, care protocols and lists of effective medication as suggested by evidence-based clinical practice [10].

In the last years, several studies on the diabetes patients’ possible benefit from the DMP have been conducted but came to inconsistent results. On the one hand, some comparative research provided evidence that DMP participation is associated with lower mortality and reduced expenses for inpatient care and medication [11] as well as improved health care processes [12]. On the other hand, there are studies which show no differences between DMP-participants and non-participants, neither to comorbidities [13] nor to outcome quality indicators such as glycated hemoglobin or blood pressure [14]. However, there is evidence that the DMP “Diabetes” impacts care provision insofar as participants perceive it more structured and patient-centered than routine care [15]. Furthermore, diabetes patients in the DMP display significantly higher ratings of health related quality of life [16].

The majority of these studies is based on data from health insurances, yet none of them is reflecting treatment and care of immigrants. To our knowledge, the study presented in this article is the first one on acceptance of and benefit from the DMP among the Turkish immigrant population with DM 2. On the basis of same process and outcome parameters we are comparing the Turkish immigrants with DM 2 with their non-immigrant counterparts in the study of Ruß-Thiel.

### 1.1. Turkish Immigrants in Germany

Over the last decades, Germany has turned into an immigration country. After World War II, the arising deficit of workforce could at first be compensated by refugees and displaced persons from Eastern European countries and migrants from the then-GDR (German Democratic Republic). In August 1961, the migration movements from Eastern Europe and the GDR suddenly stopped through the construction of the Berlin Wall and the iron curtain. As a consequence German companies started offering jobs to people from other countries. The biggest labor migration wave came from Turkey with around 830,000 persons within 15 years after the German-Turkish recruitment contract in 1961 [17]. Initially, this workers’ migration was intended to be only temporary, expecting that the workers would return to their home countries after work-life. However, the majority stayed for good. Today, three million citizens of Turkish origin are living in Germany, and the percentage of those who are older than 65 years is continually increasing [18].

### 1.2. Diabetes Mellitus Type 2 among Immigrants of Turkish Background

Studies have found an association between duration of residence and increased risk of chronic illness. Especially female migrants of older age groups seem to be affected more than any other group [19,20]. The increased risk for migrants to develop chronic diseases in older age is also expressed in high prevalence rates of diabetes. According to Salman [21], the occurrence of diabetes among migrants of Turkish origin is increasing the longer they stay in the host country. A review by Zimmet [22] revealed that migration studies indicate a change towards a “Westernized” lifestyle associated with an increase in the prevalence of diabetes type 2. This is also found by Carballo [23] arguing that the stress of migration and “new” life-style components (over-consumption of food, high consumption of alcohol and tobacco) is associated with the development of diabetes.

A more recent study on the general prevalence of diabetes indicates that the global prevalence of diabetes among adults will rise from 6.4 % in 2010 to 7.7% in the year 2030 [24]. While there are clear numbers of diabetes prevalence among the non-immigrant population, little is known about diabetes and its associated psychological and somatic impairments among elderly migrants [25]. Diabetes prevalence among Turkish migrants varies between 8% and even 15% [19,26]. Additionally, there are many elderly migrants who suffer from diabetes without knowing it [27,28]. Undiagnosed diabetes increases the risk of chronic complications and comorbidity. Thus, it enhances the importance of equal and low-threshold access to the health care system and well-adjusted, individual diabetes care for all groups in the diverse German society.

## 2. Methods

### 2.1. Study Design and Sample

The study “Health Literacy of Turkish Diabetics” was conducted between February 2008 and September 2011 and was financed by the German Federal Ministry of Education and Research (01GX0749). It explored living conditions, medical treatment and care, quality of life and health literacy of Turkish immigrants with DM 2 living in Hamburg. All subjects gave their informed consent for inclusion before they participated in the study. The study was conducted in accordance with the Declaration of Helsinki, and the protocol was approved by the Ethics Committee of the Chamber of Physicians of the federal state of Hamburg (PV3061).

From July 2008 to July 2009, 294 Turkish immigrants with DM 2 were personally interviewed by fellow countrymen with a health-related professional or educational background. The interviewers were recruited via different university departments in Hamburg and social networks of Turkish colleagues. 17 of the 18 interviewers were female; all of them speak Turkish and German fluently. They were trained intensively and continuously supervised by the research team.

The recruitment of participants in Hamburg was carried out:

(a) in cooperation with 15 doctors’ practices (10 general practitioners (GPs), five diabetologists) in Hamburg, where 130 patients were recruited consecutively, and
(b) via the interviewers’ social networks, by word-of-mouth, and via public relations, e.g., at mosques and culture clubs (164 patients).

The combination of these two modes of recruitment enabled a mostly randomized ascertainment of data. Group comparisons did not show significant socio-demographic differences between the two recruitment channels. For the following comparative analyses on benefit from and adherence to the “Diabetes 2” DMP, only those 108 participants were included who stated to be in the program.

The applied questionnaire contains questions from a cross sectional study by Ruß-Thiel [9] on benefit of and adherence to the DMP “Diabetes 2” among members of the non-immigrant population. The statutory health insurance “Gmünder Ersatzkasse” provided core data of 1300 randomly chosen insurees who were enrolled in the DMP “Diabetes 2” in 2006. These insurees were contacted, one third was not available by phone, 6% refused participation, and 702 agreed to participate and gave informed consent. Then the patients were interviewed in German. The interviewers were medical professionals working at the “ife Gesundheits-AG”, an institution providing specialist counseling to health insurances, hospitals and other institutions in the health sector. Data protection was assured during the whole process regulated by the German Federal Data Protection Act. At no point in time did the insurance influence screening, collection or analyses of data. Migrational background was not queried, as it was already known that only a minute percentage of the insurance’s members are migrants. The standardized questionnaire contains 29 care related items shown in the next paragraph.

### 2.2. Measures

For our analyses we distinguish indicators for *process quality* as well as variables indicating *outcome quality* of the DMP, used in both of the samples. If possible, responses were retrieved from the patient’s diabetes pass, if not available, it was drawn upon self-reports from the participants.

#### 2.2.1. Process Quality

For process quality we analyzed the frequencies of consultations with general practitioners and/or other relevant medical specialists. Further process quality indicators were “examination of feet”, “referrals to a podologist”, “receipt of documentation sheet” and “participation in diabetes training”.

#### 2.2.2. Outcome Quality

The following five parameters are defined as outcome quality indicators of the DMP:

- HbA_1c_
- Blood pressure
- Body weight
- Intake of fruit and vegetables
- Physical activity

Blood values are based on entries in the patient’s diabetes pass, which are made at the doctor’s practice, and only if not available on self-reports. A sum score indicates the patient’s benefit from participating in the DMP, reaching from 0 to 5. To identify to what extent DMP criteria were met, a further score, ranging from 0 to 7, was computed from the following items:

- At least two visits at the general practitioner per year
- RR ≤ 140/90 or reduced over the last months
- HbA_1c_ target is met or patient is referred to a diabetologist
- BMI < 30 or reduction of weight over the last months
- Participation in diabetes training
- 1 visit to the ophthalmologist per year
- Examination of feet or referral to podologist

BMI of the participants was calculated by the research team upon patient’s weight and height.

### 2.3. Statistical Analyses

Analyses were conducted with SPSS™. Due to the missing raw data of the non-immigrant sample of the Gmünder Ersatzkasse, only univariate procedures could be carried out. On the basis of given absolute and relative frequencies and their standard deviations, we could undertake subgroup analyses—also stratified for sex. Group differences were proved with Chi^2^-tests; in case of ordinal variables Mann-Whitney-U tests. Significance level was set at *p* < 0.05.

## 3. Results

Of the 294 Turkish immigrants under study, 108 stated to be in the DMP. The distribution of sex in the Turkish immigrant group is almost even, while there is a clear imbalance in the non-immigrant sample, foremost grounded in the specifics of the insurance’s member-structure. Turkish immigrants in our sample have been living in Germany for 33 years on average. Despite this long period, the overall proficiency of German language is rather weak. Regarding educational attainment, the majority of the Turkish immigrant sample did not go to school at all or completed elementary school only. This is also reflected in the relatively high number of illiterates in this sample (Table 1). For the non-immigrant sample there is no data on educational attainment available.

**Table 1:** Overview of Turkish immigrant *vs.* non-immigrant sample.

	Turkish Immigrant Sample (*N* = 108)	Non-Immigrant Sample (*N* = 702)
Gender female (%)	55.6	27.1
Age (M (SD))	58.1 (7.9)	63.7 (9.4)
Educational attainment (%):		
*No schooling*	22.2	not questioned
*Elementary school*	48.1	not questioned
*At least 8 years of school*	29.6	not questioned
Inability to read (%)	16.2	not questioned
Inability to write (%)	19.0	not questioned
Proficiency of German language (%)	31.7	100
Body Mass Index (M (SD))	33.2 (6.6)	only BMI groups (see Table 6)
Duration diabetes in years (M (SD))	10.6 (7.3)	not available
Years of stay in Germany (M (SD))	33.4 (7.0)	not questioned

### 3.1. Process Quality in DMP

In the structured DMP, health care is distinguished in two levels. While level one is referring to visits to the GP, the second level comprises visits to a diabetologist. In Germany, a Diabetes-DMP-patient has to be referred to a diabetologist by the GP if the target HbA_1c_ is not accomplished within six months. If there are no comorbidities or secondary diseases, the DMP requires biannual GP-consultations. Among the sample of Turkish immigrants, about 95% have seen their GP at least twice in the *past twelve months*. Slightly different, the non-immigrant patients were asked about a doctor’s visit in the *past three months*. About 96% of them responded positive to this question.

Next to the referral to the diabetologist, the DMP also requires visits to an ophthalmologist at least once a year. All of the Turkish immigrants have seen an eye specialist in the last 12 months. This is true as well for nearly all of the non-immigrant population (99.9%) (Table 2). The larger proportion in both groups visited a doctor at least once or twice per year. In total, the Turkish immigrants see the ophthalmologist more often than the non-immigrant patients.

In the DMP, the attending physician is encouraged to check the patient’s feet at least once a year for neuropathies, pulse status and/or other diabetic foot symptoms. Participants in both samples were asked whether and how often their doctor had examined their feet in the last 12 months (Table 2).

**Table 2:** Process quality: frequency of feet examination and visits to ophthalmologist (%).

	Examination of Feet		*p* *	Visits to Ophthalmologist		*p* *
	Turkish Immigrant Sample (*N* = 90)	Non-Immigrant Sample (*N* = 699)		Turkish Immigrant Sample (*N* = 102)	Non-Immigrant Sample (*N* = 654)	
<1 per year	0.0	8.8	0.014	0.0	0.1	<0.001
1–2 per year	45.6	40.9		68.6	77.1	
3–4 per year	48.9	47.0		23.5	14.2	
>4 per year	5.5	2.7		7.8	1.5	

Note: * Chi^2^-test.

In both groups, patients’ feet are checked regularly. However, about 9% of the non-immigrant population stated their feet were not examined. Another important aspect of process quality is the referral to a podologist in case of a diabetic foot (e.g., ulceration or infection or other relevant characteristics). Among the non-immigrant sample, there were 15.4% suffering from a diabetic foot, of which 43% were referred to a specialist. Among the Turkish immigrant sample, 14.3% indicated a diabetic foot, of which only 15% have been referred to a podologist.

To increase transparency and patient involvement, the DMP requires handing over copies of the documentation sheets to the patient, and to enable patients to cope better with their condition through education and training (Table 3).

**Table 3:** Process quality: receipt of documentation sheets and participation in training (%).

	Turkish Immigrant Sample (*N* = 108)	Non-Immigrant Sample	*p* *
Documentation received	88.0	70.0 (*N* = 702)	<0.001
Participation in training	91.7	65.1 (*N* = 413)	<0.001

Note: * Chi^2^-test.

In both of these process quality indicators, the Turkish diabetics have the greater share.

### 3.2. Outcome Quality in DMP

Regarding outcome quality, parameters such as HbA_1c_, blood pressure or BMI can serve as indicators for a successful participation in the program. The structured program foresees a shared decision making with the patients upon individual target values to be accomplished within one year’s time. In the interview the participants were asked to state both their current HbA_1c_ and the target value they were told by their doctor. In the sample of the non-immigrant population, about 74% were able to state their current value, which, however, is true for only 60% of the Turkish immigrant sample. Concerning the target HbA_1c_, 64% of the non-immigrants were able to recall it, while only 41% of the Turkish immigrants were effectively able to indicate this.

The average HbA_1c_ value in the non-immigrant population was 6.8%, but 8.0% in the Turkish immigrant sample. While the vast majority of the non-immigrants reported either no change or even a decrease in their HbA_1c_ values, this was only true for about 46% of the Turkish immigrants. Quite the contrary, more than the half of them experienced an increase in HbA_1c_ in the past half year (Table 4). Furthermore, it is important to mention the accumulation of responses in the category “do not know”, which is not displayed in the table: Every third diabetic of Turkish decent did not know about the course of glycated hemoglobin—this is true for only 11% among the non-immigrant sample.

In terms of blood pressure, hypertension is defined as >140 mmHg systolic and >90 mmHg diastolic, and the program also requires an agreement on target values, which should be accomplished within six months. This is why the diabetics in both samples were asked about current values as well as change in blood pressure in the past half year. A higher proportion of diabetics with Turkish origin displayed elevated values beyond 140/90 mmHg. While the bigger share of the non-immigrant sample reported stable or decreased blood pressure over the past six months, a much higher percentage of the Turkish immigrant sample had an increase. Similar to the change in HbA_1c_, more Turkish diabetics than non-immigrants did not know about the course of their blood pressure.

**Table 4:** Outcome quality: change in blood pressure and HbA_1c_ in %.

	Change in Blood Pressure		*p* *	Change in HbA_1c_		*p* *
	Turkish Immigrant Sample (*N* = 103)	Non-Immigrant Sample (*N* = 702)		Turkish Immigrant Sample (*N* = 97)	Non-Immigrant Sample (*N* = 702)	
>Than six months ago	20.4	4.0	<0.001	34.0	11.1	<0.001
No change	29.1	60.3		5.2	36.3	
<Than six months ago	15.5	31.3		24.7	41.3	
Do not know	35.0	4.4		36.1	11.3	

Note: * Chi^2^-test.

One further aim of the DMP is to optimize the patient’s lifestyle. This comprises of a healthy diet, regular physical activity and weight reduction in case of overweight. The study participants were asked about their consumption of fruit and vegetables and whether their physical activity has increased since enrollment in the DMP (Table 5).

**Table 5:** Outcome quality: healthy lifestyle in DMP (%).

	Turkish Immigrant Sample (*N* = 104)	Non-Immigrant Sample (*N* = 702)	*p* *
Physical activity ↑	39.4	34.5	0.368
Fruit & vegetables ↑	81.7	59.5	<0.001

Note: * Chi^2^-test.

While there is no difference in physical activity, significantly more Turkish immigrants report an increase in fruit and vegetable consumption.

Results for BMI were reported split according to sex (Table 6). While there are no significant differences between males in both samples, females of the Turkish immigrant sample are significantly more often represented in the higher BMI groups.

**Table 6:** Outcome quality: BMI in DMP-subgroup (%).

	Females Non-Immigrant Sample (*N* = 191)	Females Turkish Immigrant Sample (*N* = 58)	*p* *	Males Non-Immigrant Sample (*N* = 511)	Males Turkish Immigrant Sample (*N* = 48)	*p* *
<25	22.4	5.1	<0.001	14.2	8.3	0.095
25 < 30	42.1	10.2		51.9	41.7	
30 < 40	29.0	66.1		31.4	47.9	
>40	6.5	18.6		2.6	2.1	

Note: * Mann-Whitney-U-Test.

The general contentment of treatment in the DMP was also of interest. For both samples the satisfaction was high to very high: 95.9% among the Turkish immigrant group and 94.9% among the non-immigrant group.

**Figure 1:**
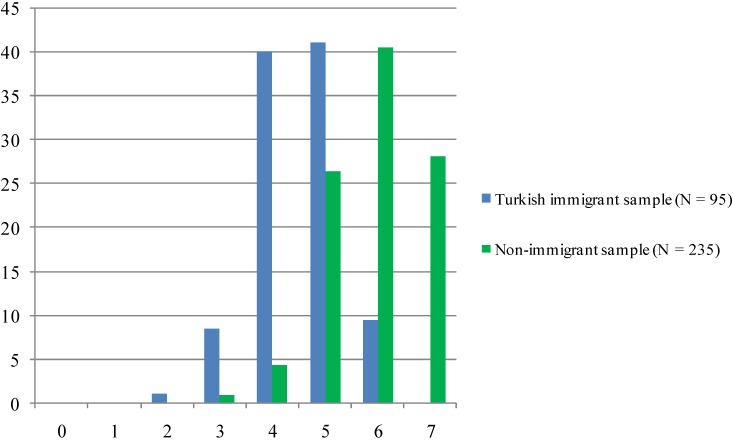
DMP criteria met (%).

Note: Sum score ranging from 0 (“no DMP criteria met”) to 7 (“all DMP criteria met”).

In order to assess the overall quality of care in the DMP, several items were totaled to obtain a score ranging from 0 to 7, representing to what extent the DMP-criteria have been met (Figure 1). The smaller sample size of N = 235 among the non-immigrant group is due to the fact that the question about participation in training was initially absent and thus added later in the ongoing survey.

While there are no participants from both samples with zero or one point only, there are clear differences in the higher ranges. No member of the Turkish immigrant sample obtained a full score, while this is true for about 28% of the non-immigrant group. In general, the non-immigrant sample achieved more points on the sum scale than diabetics of Turkish origin, whose largest share is around four and five criteria of the DMP met.

**Figure 2:**
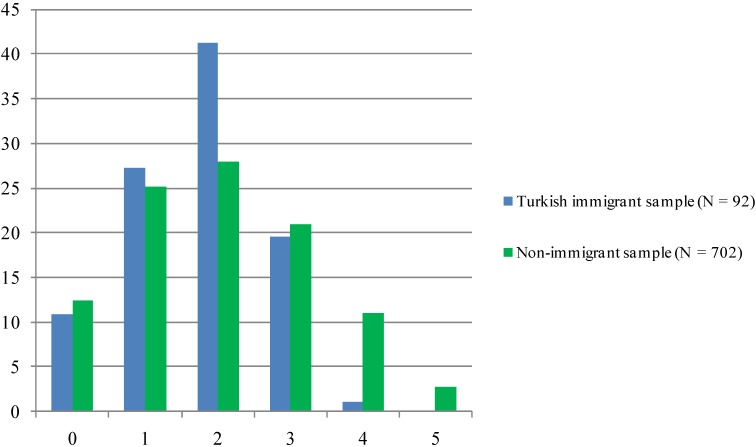
Benefit from the program (%).

Note: Sum score ranging from 0 (“no benefit”) to 5 (“maximum benefit”).

Figure 2 displays results regarding the patient’s benefit from the DMP. It is a sum score ranging from 0 to 5 which comprises parameters such a reduced HbA_1c_, blood pressure and weight as well as healthy lifestyle factors. Similar to the DMP criteria met, there were no members of the Turkish immigrant sample who obtained a full score, and also in the group that achieved 4 points the non-immigrant sample constitutes the majority. Most diabetics of Turkish origin either scored 1 or 2 points when it comes to benefit from the program.

## 4. Discussion

Our research aimed at examining the benefit from and adherence to the Diabetes 2 DMP among a sample of Turkish immigrants with diabetes in Germany. To our knowledge, this is the first study investigating whether diabetics of Turkish origin benefit equally well from the program as a group of patients with diabetes 2 that can be regarded as non-immigrant. According to DMP guidelines, several indicators for process and outcome quality of the program were observed.

Although there are obvious socio-demographic differences between the samples, we can state that the DMP-criteria are met in both groups with only slight differences. While the proportion of sex is evenly distributed among the Turkish immigrant sample, there is a preponderance of male participants in the non-immigrant sample. Beyond the specifics of the insurance’s member-structure the author also accounted for this with a higher proportion of diabetic males in the age group <70 in the sample population [9]. Participants in the Turkish immigrant sample are about five years younger on average, which can partly be explained with an earlier onset of diabetes among immigrants in Germany. This has also been found in other European countries [26], keeping in mind that our sample is random, but not population-based representative.

Mean values for the body mass index and duration since onset of diabetes were not available for the non-immigrant sample. However, descriptive results regarding BMI in the subgroups show a significantly lower BMI in the non-immigrant sample. Despite lacking representativeness, the results provide an insight into aspects of care among both DMP-members of Turkish origin and German natives. We consider it a great advantage of the study that, due to the recruiting strategy and the employment of Turkish interviewers, also those Turkish immigrants are included who cannot read or write, and are not able to understand or speak German—in this case about 50% of the participants [7].

Concerning process quality there seem to be no relevant differences between the two groups. Both are seen by their GP on a regular basis, and medical care on the second level is accomplished as required by the DMP. There is an almost equal share of diabetics who see the ophthalmologist four times a year. This is also common for consultations with the GP and other professionals. We should add that the consultation frequency corresponds with the German health care insurers’ reimbursement system for medical treatment—the budget terms are quarterly. Thus, the administrative framework additionally influences number and dates of appointments between health care professionals and patients.

Both groups fulfill the DMP requirements, but in total, the Turkish immigrant sample reported to have seen their ophthalmologist more frequently. It remains unclear, however, whether frequent visits to an ophthalmologist is due to the DMP or due to any other reason which may require regular consultations.

While a significantly greater share of the Turkish immigrants stated that their feet were checked regularly, this positive result needs to be questioned with respect for those patients with a diabetic foot syndrome. Apparently, the majority of Turkish diabetics with a foot syndrome had not been referred to the podologist, which is clearly dependent on the respective practitioner. The high amount of minor and major amputations [29] among diabetics enhances importance and necessity of interdisciplinary cooperation between different health care sectors and service providers.

In terms of transparency, significantly more Turkish immigrants have received documentation sheets and participated in training than their German counterparts. This is surprising insofar as at least a third of the Turkish patients cannot read these documents. The initial idea behind delivering relevant documents is that the patient gets an insight into his medical data and thus is strengthened to become an autonomous person in treatment and care. The DMP also intends to establish shared decision making between medical professionals and patients. However, when it comes to knowledge about individual medical parameters, the Turkish immigrant sample fared worse than the non-immigrant sample. Nevertheless, we can assume that others make use of the records and limit the risk of treatment discontinuity. This thesis is strongly supported by physicians in qualitative interviews and focus groups (not shown in the results section). They have highlighted the importance of the documents specifically for these uneducated patients when they are consulting other health care professionals. This is in particular relevant for consultations with doctors in Turkey. The patients are visiting their home country regularly for nearly a quarter of the year on average [7].

Regarding parameters of outcome quality, the majority of Turkish diabetics experienced a worsening of HbA_1c_ levels over six months prior to the interview, similar results were also obtained when asked about a change in blood pressure. Despite having participated in training significantly more often than participants of the non-immigrant sample, Turkish immigrants do not seem to benefit sufficiently from this. Of similar concern is the high share of those, who simply did not know about their HbA_1c_ or blood pressure. Regardless of the parameter in question, about every third Turkish participant was not able to answer the question about changes in these individual values. Further education and information on long-term consequences of poorly controlled blood sugar levels should be provided by medical specialists to prevent comorbidities in this vulnerable subgroup.

In contrast, the non-immigrant sample seemed to be better informed regarding the course of their personal disease-related parameters. A further reason for this discrepancy in diabetes-related knowledge and lack of benefit from specific training on behalf to the Turkish sample can be the vast differences in levels of education between the two groups. This interpretation implies the assumption that the non-immigrant sample corresponds with the educational level of the overall population in this age group in Germany. Compared to them, immigrants of Turkish decent display very low levels of education. In our study a quarter of the Turkish immigrants did not visit school at all, half of them only for four to five years, which explains the high percentage of illiterates, women more than double the men [8]. Multivariate analyses by Kofahl *et al.* [7] showed a close relation between the level of education and diabetes-related knowledge among a sample of Turkish diabetics.

Another worrisome aspect in DMP outcome parameters is BMI. As there were great differences between men and women, we displayed the results split according to sex. While females of the non-Turkish immigrant sample are mostly of normal weight or only slightly overweight (64.5% with BMI < 30), about 85% of Turkish females are obese in adiposity grades of class I to III, and also “outweigh” their male counterparts. This is against the trend among the non-immigrant population, where more females than males can be assigned to lower adiposity grades or the group of normal weight. It is difficult to find an explanation for this strongly pronounced overweight, but socially and culturally shaped dietary habits and insufficient physical activity have a strong impact [30]. In this context it is noteworthy to compare these findings with the high obesity rate of females of 15 years and older in Turkey. With 29.3% it is double the amount of Turkey’s male population (15.3%) and one of the highest in the world [31].

Although a significantly greater share of the Turkish immigrant sample stated an increase of physical activity and consumption of vegetables and fruit since enrollment in the DMP, these results have to be treated cautiously. Additionally to a diet rich in carbohydrates, vegetables and fruit have traditionally been a major element in Turkish nutritional habits. Nevertheless, an overly consumption of fruit can be contraindicated for diabetics due to their high content of rapidly cleavable fruit sugar. The inability or lack of motivation to reduce weight is worrying and underlines the fact that diabetics need further reinforcing of self-management. This empowerment also implies the integration of the patient’s social environment and aspects of emotional well-being, facets that often are neglected in the “routine” of daily medical care.

The partial performance of substantial aspects of the DMP diabetes is also reflected in both sum scores “Benefit from DMP” and “DMP criteria met”. Both groups are equally content with the treatment in general, whereas members of the non-immigrant sample benefit slightly more from the program and fulfill more relevant endpoints of the program than the Turkish immigrant sample.

Principally, and supported through the DMP, the access to migrants concerned is structurally and procedurally beneficial. The major problem in treatment and care is grounded in language barriers in the context of limited time and lack of interpreters. Some practices have staff of Turkish origin; some even Turkish speaking diabetes counsellors, but most do not. And even in this case, the mere availability of Turkish speaking staff seems not to be sufficient: One of the central findings of the Hamburg study on “Health Literacy of Turkish Diabetics” showed that the standard training programs for persons with diabetes are not suitable for Turkish immigrants with low education. To address their capabilities, there is a need for culturally adapted, text free, individualized and life contextualized training programs in the mother tongue [7,32]. In this context self-help or peer support groups could have a big potential for those concerned. However, only one person of the reviewed population reported searching for support here. Thus, it would also be important to enhance peer activities. This implies to inform about the possibility of and support through self-help groups as these are widely unknown in immigrant communities [33]. Moreover, *motivating* patients to participate in mutual and peer support seems to be even more relevant than just informing them about these opportunities.

## 5. Conclusions

When compared to the non-immigrant sample, the diabetics of Turkish decent do not benefit equally well from the DMP diabetes, but all in all results are very similar. They fulfill the criteria to a large extent and the overall contentment with treatment is very good, which underlines a positive tendency in the specific care of diabetes in this subgroup. Greater deficiencies can be seen in some substantial outcome parameters of the program. It becomes obvious that regular practices of disseminating information—either by the practitioner or via training—prove to be relatively inadequate. This enhances the necessity of a more intense support of diabetics of Turkish immigrant background throughout the DMP, incorporating cultural as well as individual specificities such as educational background and social environment in order to achieve the highest possible levels of self-management and empowerment. The DMP provides a good and feasible framework and potential for meeting these tasks and challenges.

## 6. Limitations of the Study

Some limitations have to be mentioned and considered when evaluating our findings. As the sample of Turkish immigrant diabetics is rather small and obtained from the metropolitan area of Hamburg only, it is by no means to be considered representative. Nevertheless, we think that the sampling procedure was as close as possible to representativeness under the given circumstances. Moreover, raw data for the non-immigrant sample were not available due to rights restrictions, which limit the possibilities of statistical testing and impede the generalizability of results. Nevertheless, we were able to point out first tendencies regarding Turkish immigrants’ benefit from the DMP diabetes, a topic which has—to our knowledge—not been researched before.

Both studies underlying our analyses were cross-sectional, and we cannot draw any causal conclusions. Face-to-face (alternatively via telephone in the non-immigrant sample) interviews were chosen as this method is considered most effectively to explore patients’ knowledge, behavior and attitudes. Although open questions could have given greater insight in the diabetics’ subjective experience with the DMP, standardized questionnaires were used in both studies in order to reduce time constraints.

Finally, a number of responses relied upon self-reports of the participants. Although the interviewers tried to match these responses with inscriptions in official documents (such as the diabetes pass) to increase reliability, this was not possible in every case.
